# Multicentre appraisal of amyotrophic lateral sclerosis biofluid biomarkers shows primacy of blood neurofilament light chain

**DOI:** 10.1093/braincomms/fcac029

**Published:** 2022-02-09

**Authors:** Alexander G. Thompson, Elizabeth Gray, Nick Verber, Yoana Bobeva, Vittoria Lombardi, Stephanie R. Shepheard, Ozlem Yildiz, Emily Feneberg, Lucy Farrimond, Thanuja Dharmadasa, Pamela Gray, Evan C. Edmond, Jakub Scaber, Delia Gagliardi, Janine Kirby, Thomas M. Jenkins, Pietro Fratta, Christopher J. McDermott, Sanjay G. Manohar, Kevin Talbot, Andrea Malaspina, Pamela J. Shaw, Martin R. Turner

**Affiliations:** 1 Nuffield Department of Clinical Neurosciences, University of Oxford, Oxford, UK; 2 Sheffield Institute for Translational Neuroscience, University of Sheffield, Sheffield, UK; 3 Blizard Institute, Queen Mary University of London, London, UK

**Keywords:** amyotrophic lateral sclerosis, motor neuron disease, biomarker, trial, neurofilament

## Abstract

The routine clinical integration of individualized objective markers of disease activity in those diagnosed with the neurodegenerative disorder amyotrophic lateral sclerosis is a key requirement for therapeutic development. A large, multicentre, clinic-based, longitudinal cohort was used to systematically appraise the leading candidate biofluid biomarkers in the stratification and potential therapeutic assessment of those with amyotrophic lateral sclerosis. Incident patients diagnosed with amyotrophic lateral sclerosis (*n* = 258), other neurological diseases (*n* = 80) and healthy control participants (*n* = 101), were recruited and followed at intervals of 3–6 months for up to 30 months. Cerebrospinal fluid neurofilament light chain and chitotriosidase 1 and blood neurofilament light chain, creatine kinase, ferritin, complement C3 and C4 and C-reactive protein were measured. Blood neurofilament light chain, creatine kinase, serum ferritin, C3 and cerebrospinal fluid neurofilament light chain and chitotriosidase 1 were all significantly elevated in amyotrophic lateral sclerosis patients. First-visit plasma neurofilament light chain level was additionally strongly associated with survival (hazard ratio for one standard deviation increase in log_10_ plasma neurofilament light chain 2.99, 95% confidence interval 1.65–5.41, *P* = 0.016) and rate of disability progression, independent of other prognostic factors. A small increase in level was noted within the first 12 months after reported symptom onset (slope 0.031 log_10_ units per month, 95% confidence interval 0.012–0.049, *P* = 0.006). Modelling the inclusion of plasma neurofilament light chain as a therapeutic trial outcome measure demonstrated that a significant reduction in sample size and earlier detection of disease-slowing is possible, compared with using the revised Amyotrophic Lateral Sclerosis Functional Rating Scale. This study provides strong evidence that blood neurofilament light chain levels outperform conventional measures of disease activity at the group level. The application of blood neurofilament light chain has the potential to radically reduce the duration and cost of therapeutic trials. It might also offer a first step towards the goal of more personalized objective disease activity monitoring for those living with amyotrophic lateral sclerosis.

## Teaser

In a large, multicentre study of multiple measures, Thompson and Gray *et al.* demonstrate plasma neurofilament light chain as the leading prognostic biomarker in amyotrophic lateral sclerosis. Using clinical trial modelling, they show its potential as a sensitive outcome measure to more rapidly assess candidate therapeutics.

## Graphical Abstract

**Graphical Abstract:**
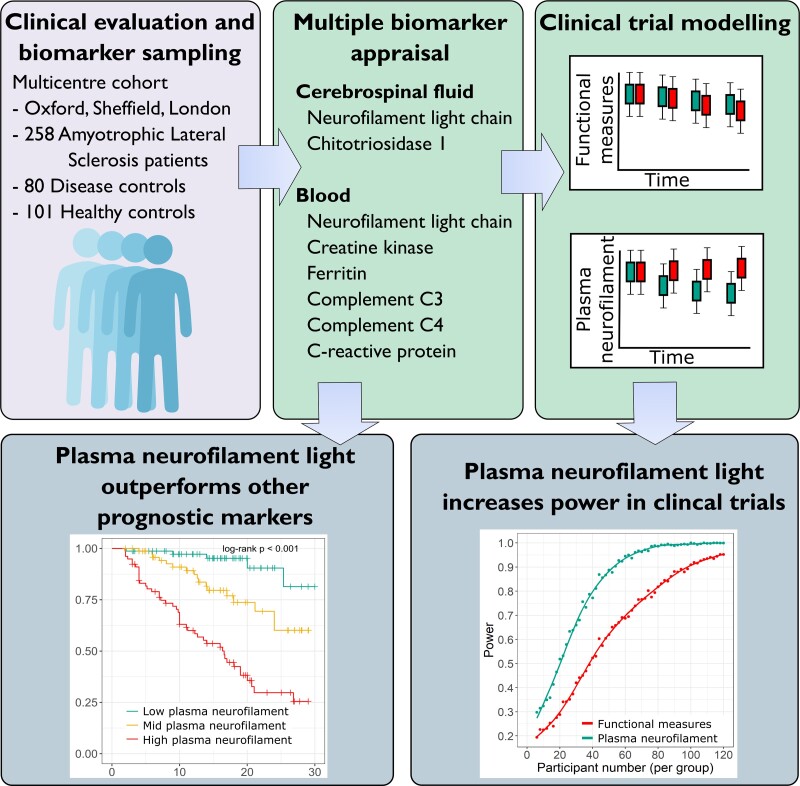

## Introduction

Amyotrophic lateral sclerosis (ALS) is a neurodegenerative disorder associated with loss of motor neuronal system integrity, resulting in progressive muscle weakness for which there is no highly effective therapy. Although the median survival is <3 years from symptom onset, there is substantial clinical heterogeneity and a variable rate of disability progression.^1^ This makes therapeutic trial design challenging. Patient survival and the rate of decline of the revised ALS Functional Rating Scale (ALSFRS-R) are commonly used outcome measures in the absence of biomarkers, necessitating costly trials, with large group sizes and lengthy follow-up to detect efficacy.^2^

The complex genetic landscape of ALS implies that motor neuron degeneration is the final common pathway of multiple upstream defects, requiring a much more individualized approach to future therapy and potentially further compounding the issue of clinical trial power.^3^ The Airlie House ALS Clinical Trials guidelines recognized that trial designs integrating objective markers of disease activity are a key priority for therapy development.^4^

Neurofilaments are axonal cytoskeletal proteins found in the CSF and blood in a wide range of central nervous system disorders, with levels broadly linked to the rate of disease progression.^5^ In ALS, studies spanning more than two decades have correlated neurofilament light chain (NFL) levels with the rate of both disability accrual, as measured by the decline in the ALSFRS-R, and with overall survival.^6–8^ Individual longitudinal CSF NFL levels appeared relatively stable over time,^9^ making it a candidate pharmacodynamic marker of rate of disease progression in therapeutic trials.^10^

Chitotriosidase 1 (CHIT1) reflects microglial activity, and is detectable at increased levels in the CSF of ALS patients, also correlating with disease progression rate (PR) and survival.^11,12^ Several analytes measurable using routine clinical assays have also shown potential value as biomarkers in ALS, including acute phase proteins: C-reactive protein (CRP), complement, ferritin,^13–17^ and the skeletal muscle marker creatine kinase (CK).^18,19^

We sought to appraise these leading candidate biomarkers in a large, longitudinal cohort of individuals diagnosed with ALS, specifically considering the case for routine integration into clinical care, for prognostic stratification and as supportive markers of therapeutic efficacy.

## Materials and methods

### Participants and sampling

A Multicentre Biomarker Resource Strategy In ALS (‘AMBRoSIA’) is a longitudinal cohort based on individuals attending three UK tertiary ALS referral clinics (in Oxford, Sheffield and London). Participants were diagnosed by neurologists specializing in the diagnosis and treatment of ALS (K.T., M.R.T., P.J.S., C.J.M., T.M.J., A.M.). All participants provided written informed consent. ALS patients were offered longitudinal assessments. Patients with other conditions and a group of healthy individuals recruited from spouses and friends of clinic attendees were sampled at a single timepoint. Diagnoses of those with other conditions is summarised in Supplementary Table 7. Recruitment commenced in June 2017 with a censorship date for survival analyses of 01/02/2020. Ethical approval for the study was obtained from London—South East Research Ethics Committee (16/LO/2136).

CSF samples from lumbar puncture went directly into polypropylene tubes. Venous blood was collected using the BD Vacutainer Safety-Lock set into serum separator tubes, EDTA or lithium heparin tubes depending on the biomarker being analysed. Blood and CSF samples were centrifuged at 3500 rpm for 10 min at 4°C within 1 h of sampling and stored in vapour phase nitrogen until measurement. Longitudinal samples were obtained during routine follow-up clinic visits at intervals of 3–6 months. ALS patient deaths were noted as part of routine healthcare record updating.

Clinical measures were obtained on the same day as biofluid sampling. Symptom onset was defined as the date and region of first muscle weakness. Physical disability was assessed using the ALSFRS-R and forced vital capacity (FVC). The Edinburgh Cognitive and Behavioural ALS Screen (ECAS) was undertaken at the first visit by researchers trained in its use. A baseline disease progression rate (PR, points/month) was calculated using the formula: (48-ALSFRS-R score)/(months from symptom onset at first sampling). A longitudinal rate of change of the ALSFRS-R (delta FRS, D-FRS) was calculated by subtracting the last recorded ALSFRS-R score from the baseline ALSFRS-R score and dividing by the interval between visits (in months).^20^

### Biochemical assays

All assays were performed in duplicate according to manufacturers’ instructions. Samples were fully thawed on ice following removal from vapour phase nitrogen storage prior to measurement of analytes. Measurements of NFL in CSF and plasma were performed using the Meso Scale Discovery R-PLEX electrochemiluminescence platform by the Oxford, London and Sheffield teams, with inter-site sample exchange to assess inter-laboratory variation. CSF CHIT1 was measured by Oxford using the CircuLex ELISA. Plasma NFL levels were measured in aliquots of 10 identical samples across the three centres {inter-site median coefficient of variation (CV) 12.1% [interquartile range (IQR) 8.3–17.4%]}. Plasma NFL intra-assay and inter-assay CVs were median 2.8% (IQR 1.1–5.4%) and median 9.3% (IQR 7.7–12.3%), respectively. CSF NFL intra-assay and inter-assay CVs were median 1.4% (IQR 0.7–2.9%) and median 3.1% (IQR 1.4–5.2%), respectively. CHIT1 intra-assay and inter-assay CVs were median 3.7% (IQR 1.3–7.3%) and 4.9% (IQR 4.0–20.1%).

Ferritin, CK and CRP were measured by Clinical Biochemistry Laboratories at Oxford University Hospitals NHS Foundation Trust, Sheffield Teaching Hospitals NHS Foundation Trust and Barts Health NHS Trust, London. Further details of clinical laboratory assays are described in Supplementary Methods.

### Statistical analysis

Statistical analysis was performed in R. Analyte levels followed a log-normal distribution, hence cross-sectional analysis was performed using log_10_-transformed values. For CRP, which included zero values, 0.01 was added to all values to avoid infinite log-transformed values whilst maintaining a normal distribution on inspection of quantile–quantile plots. Due to differences in age and sex between groups, cross-sectional analysis of analyte levels was performed using analysis of covariance (ANCOVA) adjusting for age, sex and recruitment site with *post hoc* pairwise ANCOVA comparing levels in ALS and healthy control or disease control participants, adjusting for age, sex and recruitment site. The resulting *P*-values were false discovery rate (FDR)-adjusted across all analytes.

Associations between different analyte levels and with clinical variables were examined using Pearson correlation and multivariate linear regression of log-transformed analyte levels. PR and D-FRS were log_10_ transformed. *P*-values for analyte coefficients were FDR-adjusted across all analytes.

Survival analysis was performed using a log-rank test by analyte tertile and Cox proportional hazards modelling. Analytes and PR were log_10_ transformed, centred and scaled prior to analysis. The resulting hazard ratios (HRs), therefore, reflect a one standard deviation (SD) rise in log_10_ biomarker level or PR. Missing data were imputed for the blood (10.1%) and CSF (14.6%) datasets using multiple imputation by chained equations with 50 iterations and 100 imputations, with Nelson–Aalen estimates of cumulative survival.^21^ Clinical variables included were those previously associated with survival that were available in this dataset: site of symptom onset, age at symptom onset, latency from symptom onset and PR at first visit, as reported in the European Network for the Cure of ALS survival model.^1^ The ALS-specific sub-score of the ECAS was included as a continuous covariate to consider the adverse prognostic influence of some aspects of cognitive impairment on prognosis in ALS. Parameter estimates were combined across imputations using Rubin’s rules. The resulting *P*-values were FDR-adjusted across all analytes. Comparison of model fit was made using the Akaike information criterion (AIC) including and excluding plasma NFL.

Longitudinal analysis was performed for participants with two or more timepoint measurements using random slope, random intercept linear mixed-effects models. Individual participants were specified as random effects using an unstructured covariance matrix, with degrees of freedom as described by Pinheiro and Bates.^22^ Separate models were created for both disease duration from symptom onset and duration from baseline study visit. Due to the early rises in analyte levels previously identified in longitudinal analysis of neurofilament and chitinase proteins,^23,24^ separate models were constructed for measurements within the first 12 months from symptom onset, after 12 months, and for a model including all timepoints. The resulting *P*-values were FDR-adjusted across all longitudinal models for all analytes.

Clinical trial simulations were performed using longitudinal linear mixed-effects models. Plasma NFL levels were log transformed; 48-ALSFRS-R was normalized using a Box-Cox transformation. Models of transformed plasma NFL were constructed with fixed effects for log(PR) and time from baseline, and models of transformed ALSFRS-R were constructed with fixed effects for log(PR) and time from baseline, both with per-participant intercept and slope random effects. Baseline PR and latency to enrolment were simulated from a bivariate normal distribution according to the study dataset. Since PR has been robustly associated with survival, ongoing disease progression and plasma NFL, treatment effects were varied by proportionally reducing untransformed PR using an exponential decay function, with a corresponding effect on longitudinal ALSFRS-R and plasma NFL. Attrition of 5% per 2-monthly visit was imputed using the last observation carried forwards. Data simulated for a 6-month clinical trial were analysed using mixed models for repeated measures, with an equal number of participants allocated to placebo (i.e. proportional treatment reduction of zero) or treated groups, varying *n* from 6 to 120 participants per group in increments of two participants with 1000 replicated trials for each value of *n* and treatment effect from 0.1 to 0.5 (i.e. maximum of a 50% reduction in PR) in increments of 0.1. Power was estimated for *α* = 0.05 at each value of *n*.

Since recent ALS therapy trials tend to restrict enrolment to patients within 24 months of symptom onset, the base models upon which simulations were performed were constructed using the first 8 months of data for participants enrolled within 24 months of symptom onset (*n* = 63 participants with longitudinal data). Full details of the simulation approach are detailed in the Supplementary Methods.

### Availability of data

Anonymised data are available from the corresponding author by reasonable request.

## Results

### Demographic data

Demographic data are given in Table 1.

**Table 1:** Clinical and demographic characteristics

	Blood				CSF			
	ALS	HC	DC	*P*	ALS	HC	DC	*P*
Visit 1 *n*	258	101	80	–	111	22	38	–
Visit 2 *n*	120	–	–	–	36	–	–	–
Visit 3 *n*	61	–	–	–	13	–	–	–
Visit 4 *n*	30	–	–	–	–	–	–	–
Visit 5 *n*	14	–	–	–	–	–	–	–
Visit 6 *n*	7	–	–	–	–	–	–	–
Visit 7 *n*	5	–	–	–	–	–	–	–
Visit 8 *n*	2	–	–	–	–	–	–	–
Visit 9 *n*	1	–	–	–	–	–	–	–
Male, *n* (%)	171 (66.54)	32 (31.68)	51 (63.75)	<0.001^a^	74 (66.67)	8 (36.36)	21 (55.26)	0.023^b^
Age at sampling, mean ± SD (years)	62.3 ± 11.8	55.2 ± 13.2	55.1 ± 18.2	<0.001^c^	61 ± 12.2	46.9 ± 15.4	53.9 ± 20.2	<0.001^d^
Age at symptom onset, mean ± SD (years)	59.7 ± 12	–	–	–	59 ± 12.6	–	–	–
Disease PR, median [IQR] (points per month)	0.4 [0.2–0.8]	–	–	–	0.4 [0.2–0.9]	–	–	–
Bulbar, *n* (%)	60 (24.1)	–	–	–	23 (21.5)	–	–	–
Deaths, *n* (%)	70 (27.7)	–	–	–	33 (31.7)	–	–	–
Follow-up duration, median [IQR] (months)	14.6 [9–19.2]	–	–	–	13.5 [7–18.1]	–	–	–
ALS-specific ECAS score, median [IQR]	85 [76.2–90]	–	–	–	85 [77.5–90]	–	–	–

ALS, amyotrophic lateral sclerosis; HC, healthy control; DC, disease control.

^a^ *χ* ^2^-test with *post hoc* pairwise Fisher exact test; ALS-HC *P* < 0.001, ALS-DC not significant, HC-DC *P* < 0.001.

^b^ *χ* ^2^-test with *post hoc* pairwise Fisher exact test; ALS-HC *P* = 0.044, ALS-DC not significant, HC-DC not significant.

^c^ Kruskal–Wallis H-test with *post hoc* pairwise Mann–Whitney U-test; ALS-HC *P* < 0.001, ALS-DC *P* = 0.010, HC-DC not significant.

^d^ Kruskal–Wallis H-test with *post hoc* pairwise Mann–Whitney U-test; ALS-HC *P* < 0.001, ALS-DC not significant, HC-DC not significant.

### Cross-sectional biomarkers raised in ALS

**Figure 1:**
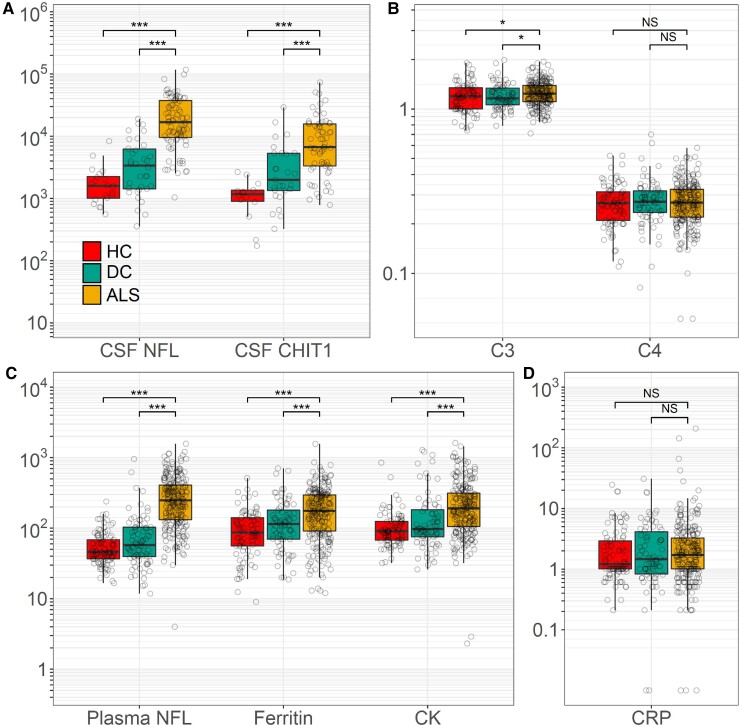
**Cross-sectional biomarker levels.** (**A**) Cross-sectional levels of CSF NFL and CHIT1 (pg/ml). CSF levels of NFL and CHIT1 were elevated in the first-visit CSF samples of ALS patients compared with healthy (NFL *P* < 0.0001, CHIT1 *P* < 0.0001) and disease controls (NFL *P* < 0.0001, CHIT1 *P* < 0.0001). (**C**) Cross-sectional levels of plasma NFL (pg/ml), serum ferritin (μg/L), plasma CK (IU/l) and serum C3 (g/l) were elevated in the first-visit CSF samples of ALS patients compared with healthy (for NFL *P* < 0.0001, ferritin *P* < 0.0001, CK *P* < 0.0001, C3) and disease controls (NFL *P* < 0.0001, ferritin *P* = 0.003, CK *P* < 0.001, C3). Serum C4 (g/l) (**B**) and plasma CRP (mg/l) (**D**) levels were similar between ALS compared with healthy controls (CRP *P* = 0.612, C3 *P* = 0.064, C4 *P* = 0.861) and disease controls (CRP *P* = 0.883, C3 *P* = 0.209, C4 *P* = 0.899). All data are displayed as median ± IQR; *P*-values given for ANCOVA of log-transformed analyte levels, controlling for age, sex and study site. ALS, amyotrophic lateral sclerosis; DC, disease control; HC, healthy control; NFL, neurofilament light chain; CHIT1, chitotriosidase 1; CK, creatine kinase; CRP, C-reactive protein; C3, complement C3 protein; C4, complement C4 protein. *P < 0.05; ***P < 0.001, NS, not significant.

Levels of NFL and CHIT1 were elevated in the first-visit CSF samples of patients with ALS compared with healthy controls (NFL mean 13 994.7 pg/ml ALS, 1729.2 pg/ml healthy control, *P* < 0.001; CHIT1 ALS 6869.4 pg/ml, healthy control 843.5 pg/ml, *P* < 0.001) and disease controls (NFL 2742.9 pg/ml, *P* < 0.001; CHIT1 2194.1 g/ml, *P* < 0.001). Levels of plasma NFL, plasma CK, serum ferritin and complement C3 protein (C3) were elevated in first-visit samples of ALS patients compared with healthy (NFL ALS 216.70 pg/ml, healthy control 50.2 pg/ml, *P* < 0.0001; CK ALS 191.3 IU/l, healthy control 101.60 IU/l, *P* < 0.001; ferritin ALS 121.96 µg/l, healthy control 79.7 µg/l, *P* < 0.001; C3 ALS 1.3 g/l, healthy control 1.2 g/l, *P* = 0.021) and disease controls (NFL 65.9 pg/ml, *P* < 0.0001; CK 125.8 IU/l, *P* < 0.001; ferritin 91.1 µg/l, *P* = 0.015; C3 1.2 g/l, *P* = 0.038). Plasma CRP and serum complement C4 protein (C4) levels were similar between ALS, healthy (CRP *P* = 0.612, C4 *P* = 0.671) and disease controls (CRP *P* = 0.957, C4 *P* = 0.957) (Fig. 1).

### Relationship between-analyte levels and clinical variables

#### Progression rate

Higher levels of plasma NFL, CSF NFL and CSF CHIT1 were moderately correlated with higher PR (plasma NFL Pearson’s *r* = 0.48, *P* < 0.001; CSF NFL *r* = 0.46, *P* < 0.001; CSF CHIT *r* = 0.42, *P* = 0.005) and with D-FRS (plasma NFL *r* = 0.59, *P* < 0.001; CSF NFL *r* = 0.60, *P* < 0.001; CSF CHIT 0.48, *P* = 0.012; Supplementary Table 1 and Fig. 1). In multiple linear regression models controlling for age at sampling, site of symptom onset and sex, results for both PR (plasma NFL slope = 0.58, *P* < 0.001; CSF NFL slope = 0.45, *P* < 0.001; CSF CHIT slope = 0.37, *P* = 0.023) and for D-FRS (plasma NFL slope = 0.26, *P* < 0.001; CSF NFL slope = 0.23, *P* < 0.001; CSF CHIT slope = 0.14, *P* = 0.027) remained significant (Supplementary Tables 2 and 3).

Weak correlation was noted between serum C3 and C4 and PR (C3 *r* = 0.16, *P* = 0.045; C4 *r* = 0.21, *P* = 0.008), with a significant relationship also demonstrated in multiple linear regression models controlling for age at sampling, site of symptom onset and sex (C3 slope = 0.947, *P* = 0.056; C4 slope = 0.55, *P* = 0.048; Supplementary Fig. 1 and Tables 1–3).

#### Other clinical variables

Results of correlation and multiple linear regression models for other clinical variables are given in Supplementary Fig. 1 and Tables 1, 4, 5 and 6; relationships that were significant after correcting for multiple comparisons are outlined below.

Levels of plasma NFL and serum C4 were weakly negatively correlated with ALSFRS-R (plasma NFL *r* = −0.16, *P* = 0.032; C4 *r* = −0.213, *P* = 0.021). Plasma NFL and serum C4 were also negatively associated with ALSFRS-R by multiple linear regression (plasma NFL slope = −3.87, *P* = 0.050; C4 slope = −9.23, *P* = 0.050). Levels of CSF NFL, plasma CRP and serum C3were negatively correlated with first-visit ALSFRS-R (CSF NFL *r* = −0.26, *P* = 0.032; CRP *r* = −0.17, *P* = 0.032; C3 *r* = −0.16, *P* = 0.032); plasma CK levels were positively correlated with ALSFRS-R (*r* = 0.18, *P* = 0.032). In multiple linear regression models controlling for age at sampling, site of symptom onset and sex, no significant associations with plasma NFL, CRP and CK, and serum C3 with ALSFRS-R were found.

Plasma CRP and serum C3 levels were negatively correlated with FVC (CRP *r* = −0.21, *P* = 0.047; C3 *r* = −0.23, *P* = 0.034) but in multiple linear regression models controlling for age of sampling, site of symptom onset and sex, no relationship between plasma CRP or serum CP and FVC was identified.

Levels of C3 and CRP were weakly negatively correlated with FVC (C3 *r* = −0.23, *P* = 0.034; CRP *r* = −0.21, *P* = 0.047); no associations were observed for ALS-specific ECAS score (Supplementary Tables 1, 5 and 6).

### Correlations between biochemical variables

Between-analyte correlations are given in Supplementary Fig. 2. Levels of plasma and CSF NFL were strongly correlated (*r* = 0.86, *P* < 0.001), followed by CSF CHIT1 with CSF NFL (*r* = 0.75, *P* < 0.001) and plasma NFL (*r* = 0.68, *P* < 0.001). Levels of serum C3 and C4 were moderately correlated (*r* = 0.43, *P* < 0.001) and with plasma CRP (C3 *r* = 0.37, *P* < 0.0001; C4 *r* = 0.32, *P* < 0.001). Serum ferritin was weakly correlated with serum C3 (*r* = 0.22, *P* = 0.036).

### Survival analyses

**Figure 2:**
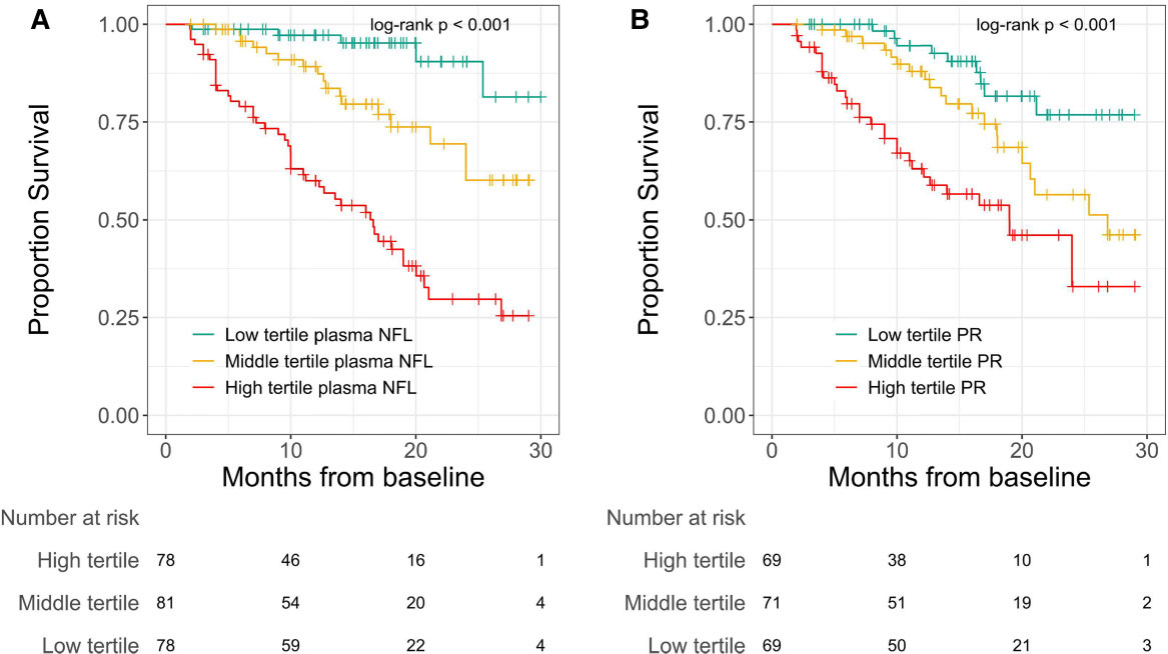
**Survival analysis.** Kaplan–Meier survival estimates for patients with ALS stratified by tertile of (**A**) plasma NFL and (**B**) baseline PR (participants with data for plasma NFL, *n* = 237) with log-rank test. *P*-value indicated for NFL or PR tertiles. NFL, neurofilament light chain; PR, progression rate; ALS, amyotrophic lateral sclerosis.

In Cox proportional hazards models incorporating clinical variables previously associated with survival along with all blood analytes measured, plasma NFL was the only variable independently associated with shortened survival (HR for one SD rise in log_10_ NFL = 3.0, 95% confidence interval 1.7–5.4, *P* = 0.016). A model incorporating clinical variables with plasma NFL and CSF analytes yielded no significant associations (Table 2), though model fit as measured by AIC was improved with the inclusion of plasma NFL. Kaplan–Meier curves for PR and plasma NFL with univariate analysis by log-rank test are given in Fig. 2.

**Table 2:** Cox proportional hazards modelling

	Blood (*n* = 248)			CSF (*n* = 102)		
	HR [95% confidence interval]	*P*	Adj *P*	HR [95% confidence interval]	*P*	Adj *P*
Age of onset	1.39 [0.96–2.02]	0.087	0.347	1.04 [0.63–1.71]	0.880	0.880
FVC	0.62 [0.40–0.98]	0.051	0.308	0.77 [0.44–1.35]	0.382	0.880
Spinal onset	1.39 [0.96–2.02]	0.087	0.347	1.04 [0.63–1.71]	0.880	0.880
PR	0.77 [0.38–1.57]	0.483	0.828	1.43 [0.94–2.17]	0.117	0.528
ALS-specific ECAS score	1.16 [0.71–1.89]	0.572	0.858	1.17 [0.57–2.41]	0.680	0.880
Latency from symptom onset	0.74 [0.50–1.11]	0.159	0.477	0.87 [0.49–1.54]	0.643	0.880
Plasma NFL	**2.99 [1.65–5.41]**	**0**.**001**	**0**.**016**	5.13 [1.05–25.19]	0.062	0.528
CSF NFL	–	–	–	1.12 [0.30–4.16]	0.871	0.880
CSF CHIT1	–	–	–	0.91 [0.34–2.41]	0.853	0.880
C3	1.38 [0.70–2.69]	0.367	0.735	–	–	–
C4	0.97 [0.50–1.88]	0.918	0.918	–	–	–
CRP	0.96 [0.54–1.68]	0.876	0.918	–	–	–
Ferritin	0.91 [0.51–1.61]	0.741	0.918	–	–	–
CK	0.78 [0.50–1.20]	0.268	0.644	–	–	–

Imputed datasets, incorporating available clinical parameters with all blood biochemical analytes or CSF analytes with plasma NFL. These indicate a significant independent association for plasma NFL in models of blood analytes, but no significant associations for CSF analytes with plasma NFL. Bold values indicate adjusted *P*-value <0.05.

HR, hazard ratio; CI, confidence interval; FVC, forced vital capacity; PR, progression rate; ALS, amyotrophic lateral sclerosis; ECAS, Edinburgh Cognitive and Behavioural ALS Screen; NFL, neurofilament light chain; CHIT1, chitotriosidase 1; C3, complement C3 protein; C4, complement C4 protein; CRP, C-reactive protein; CK, creatine kinase.

### Longitudinal analysis

#### CSF and plasma NFL

**Figure 3:**
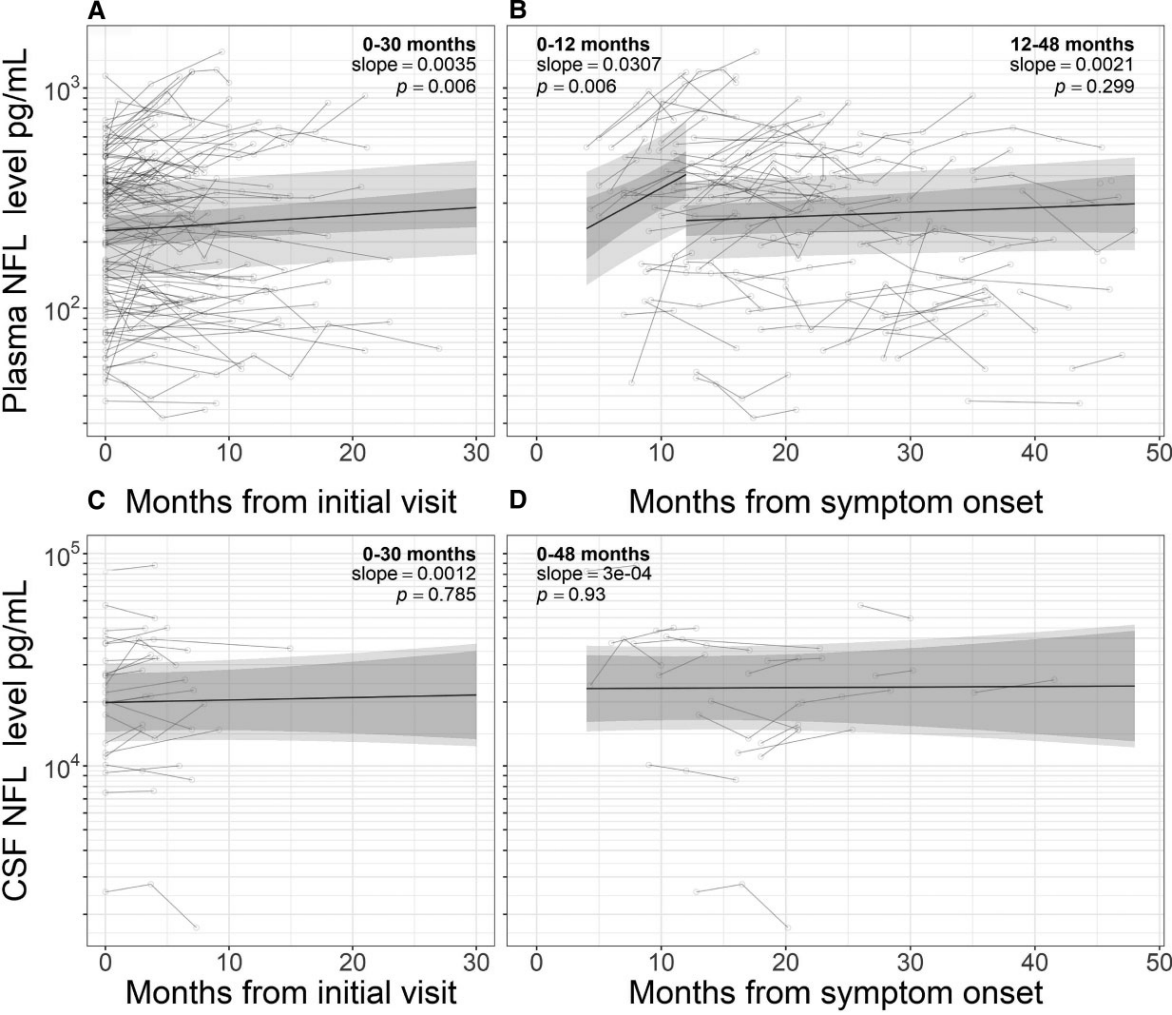
**Longitudinal analysis.** Longitudinal analysis of NFL levels in plasma (**A** and **B**) and CSF (**C** and **D**) of patients with ALS. Linear mixed-effects models were fitted to data from baseline visit (**A** and **C**) and from symptom onset (**B** and **D**). Separate models of plasma neurofilament from symptom onset were constructed for samples taken within 12 months of symptom onset and those 12–48 months from symptom onset. Longitudinal data for other analytes are given in Supplementary Fig. 4. NFL, neurofilament light chain; ALS, amyotrophic lateral sclerosis.

Longitudinal CSF NFL levels remained stable when modelled from initial visit (slope 0.0012 log units/month, *P* = 0.687) and symptom onset (slope 0.000, *P* = 0.930). A small increase was noted in plasma NFL levels measured from baseline visit (slope 0.004, *P* = 0.006; Fig. 3). Measured from symptom onset, there was a sharp rise in the first 12 months (slope 0.031, *P* = 0.006) though levels did not change significantly beyond this (12–48 months slope 0.002, *P* = 0.299).

#### Other analytes

CSF CHIT1 level slightly increased longitudinally when modelled both from initial visit (slope 0.013, *P* = 0.018) and symptom onset (slope 0.012, *P* = 0.038; Supplementary Fig. 3). Longitudinal increases in serum C4 were observed when modelled from baseline visit (slope 0.004, *P* = 0.014; Supplementary Fig. 4).

### Modelling effect of including plasma NFL in therapeutic trials

**Figure 4:**
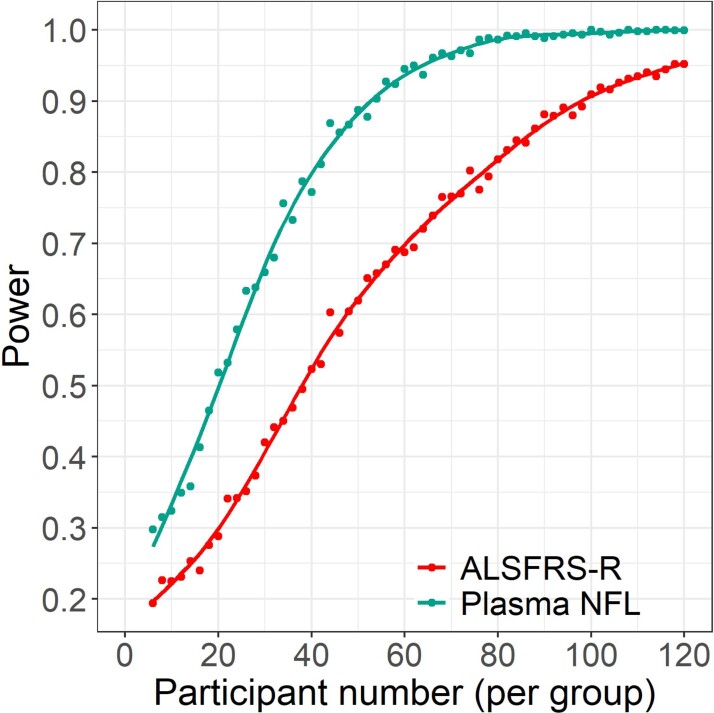
**Power simulations for randomised placebo-controlled trial.** Illustrating a treatment effect corresponding to a 40% reduction in baseline PR, comparing difference in ALSFRS-R change (red) or plasma NFL (green) between groups using mixed models for repeated measures, demonstrating improved statistical power (1 − *β*) for a given sample size when using plasma NFL as an outcome measure. Points indicate simulated power at given sample size for 1000 iterations with *α* = 0.05. ALSFRS-R, revised ALS Functional Rating Scale score; NFL, neurofilament light chain; *n*, number of participants per group.

In order to evaluate the performance of plasma NFL as a clinical trial outcome measure, trial simulations were conducted based on data collected during AMBRoSIA to estimate the power to detect a significant difference between groups, *α* = 0.05, for varying numbers of participants using either plasma NFL or ALSFRS-R as an outcome measure. Data for a treatment equivalent to a proportional reduction in PR of 0.4, reflecting a median reduction in ALSFRS-R decline at 6 months of 1.79 points (35% of untreated decline, comparable with the reported proportional effect of edaravone) and an equivalent reduction of plasma NFL are shown in Fig. 4. The estimated sample size to achieve 80% power for this effect size is ∼75 participants per group using the ALSFRS-R as an outcome measure. To provide 80% power using plasma NFL as an outcome measure would require ∼40 participants per group.

## Discussion

This large, multicentre, longitudinal, multi-modal biofluid biomarker cohort study appraised the performance of a range of leading candidate blood and CSF biomarkers in those diagnosed with ALS. This demonstrated the superior performance of neurofilament levels over other biomarkers in terms of association with key measures of the aggressiveness of ALS, specifically the rate of progression of functional decline as measured by the ALSFRS-R and survival. Interestingly, our analysis suggests that plasma NFL, but not CSF NFL, was associated with survival in multivariate models controlling for other biochemical and clinical parameters. Our clinical trial modelling indicates that measurement of plasma neurofilament is a more sensitive reflection of the underlying disease process than measurement of the ALSFRS-R.

This study was not designed to answer the question of a diagnostic role for NFL or any other biomarker in ALS. Although several markers were significantly raised in the ALS group compared with both disease and healthy control groups, as previously demonstrated for NFL,^25^ the diagnosis of ALS is still largely based on the clinical narrative, examination findings and supportive electromyography.

This study confirmed that NFL levels reflect the rate of disability progression as well as the overall survival of ALS patients. Notably, this study indicated that CSF NFL did not provide any significant advantage over plasma levels (albeit based on a smaller longitudinal cohort in this analysis), making measurement a practical option for the routine clinic environment. NFL is considered to reflect disease activity, rather than being a marker of disease progression based on absolute neuronal loss. The rate of disability progression for an individual with ALS is largely stable throughout the disease course and longitudinal analysis of CSF NFL was consistent with the stability of levels seen in a previous study.^9,26^ Plasma NFL showed a previously observed slow rise in level,^27^ which may be driven by patients with a shorter latency from the first symptom to the first assessment. Greater variability in survival prediction has been noted in ALSFRS-R modelling of patient-reported symptom onset date in such individuals.^26^ This suggests additional timepoint NFL level measurement may be needed for those within 12 months of first symptom onset.

Although CSF CHIT1 level demonstrated convincing association with measures of the rate of disease progression, it did not show an association with survival independent of NFL, in line with previous observations.^28^ These data still provide support for a more nuanced role in assessing therapies that specifically target neuroinflammatory mechanisms.

Plasma CK, serum ferritin and, to a lesser extent, C3 levels were higher in ALS patients than both disease and healthy control groups but did not add independent prognostic information. Levels of serum C4 and plasma CRP did not differ significantly between patients and control groups and showed inconsistent correlation with clinical parameters and survival, in keeping with the variable results previously published.^16,29,30^

Rapid falls in neurofilament levels have been observed following the initiation of effective treatment in both HIV neurocognitive disorder and multiple sclerosis.^31,32^ Consistent reduction in neurofilament levels has also recently been shown in trials of antisense oligonucleotide therapy in patients harbouring ALS-causing *SOD1* mutations, providing an early suggestion that similar changes will occur with effective ALS treatment.^10^ Our modelling suggested that using plasma NFL in place of the ALSFRS-R in clinical trials offers increased power to detect treatment effects in smaller group sizes. This effect is most relevant when treatment effects are expected to be small, which is the case for the only licenced disease-modifying ALS treatments to date, and might help explain why significant reductions in neurofilament levels observed with antisense therapy have not been accompanied by significant improvement in disability progression. The performance of NFL compared with the ALSFRS-R may be attributable to the fact that NFL levels reflect disease activity within a narrower time window compared with the accrual of disability.

The clinical trial simulation is predicated on several assumptions. Firstly, it assumes that an effective ALS treatment would have an effect on the plasma neurofilament level. For disease-modifying treatments aiming to slow the rate of motor neuron degeneration, this assumption is reasonable, given that plasma neurofilament levels fall following the initiation of treatment in other neurological diseases.

It has also been assumed that the effect of treatment on neurofilament levels and the rate of functional decline would be related and similar in time course; this assumption is currently impossible to test due to the lack of effective treatment. It is also assumed that treatment would not significantly improve function, only halt its decline. It is highly unlikely that even very effective treatment could reverse the neuronal loss underlying the symptoms of ALS, though recovery of dysfunctional cells and reinnervation might lead to a degree of recovery which would underestimate the effect of treatment on the ALSFRS-R score; this is likely to have a modest impact on this analysis, but to avoid underestimating this effect in highly effective treatments we have limited analysis to treatments with a smaller effect. Although the rate of decline of the ALSFRS-R in simulations is slightly slower than that of the large-scale Pooled Resource Open-Access Clinical Trial (PRO-ACT) database and the edaravone study, which might impact the power to detect a difference in ALSFRS-R,^33,34^ this would not be expected to exceed the improvement in power observed using plasma NFL.

Our analysis offers support for the routine inclusion of plasma NFL in future therapeutic trials in ALS. It is recognized that the primary goals of disease-modifying treatment in ALS are to abrogate the accrual of disability and prolong survival, so that measurement of plasma NFL might support, but not currently replace, established clinical outcome measures in phase III trials. There is potential for a very valuable role in interim analyses, and prioritization of neurofilaments as an outcome measure at an earlier stage of drug development might enable the use of very small cohorts as a means to assess multiple drug targets in early-phase studies to determine which are most appropriate to take forward for the definitive phase III trials. It is recognized that therapeutic strategies not influencing cellular neurodegeneration, for example skeletal muscle calcium sensitizers,^35^ would not be expected to lead to changes in neurofilament levels, though still have potential to influence survival and disability, and it is noted that one small trial has demonstrated differences in disease progression using a neuroprotective strategy that was not accompanied by a fall in neurofilament levels, though this finding awaits confirmation in larger studies.^36^ Conversely, some interventions might influence neurofilament levels, such as directly targeting neurofilament turnover, without resulting in a change in the rate of disability accrual or survival.

Beyond its value for prognostic stratification at group level, the introduction of routine blood NFL measurement as an objective estimate of disease activity for the individual living with ALS might offer a first step towards more personalized medicine.

## Supplementary Material

fcac029_Supplementary_Data

## Abbreviations

AIC =: Akaike information criterion
ALS =: amyotrophic lateral sclerosis
ALSFRS-R =: Amyotrophic Lateral Sclerosis Functional Rating Scale
AMBRoSIA =: A Multicentre Biomarker Resource Strategy In ALS
ANCOVA =: analysis of covariance
C3 =: complement C3 protein
C4 =: complement C4 protein
CI =: confidence interval
CHIT1 =: chitotriosidase 1
CK =: creatine kinase
CRP =: C-reactive protein
CV =: coefficient of variation
D-FRS =: rate of change of ALSFRS-R
ECAS =: Edinburgh Cognitive and Behavioural ALS Screen
FDR =: false discovery rate
FVC =: forced vital capacity
HR =: hazard ratio
IQR =: interquartile range
PR =: progression rate
PRO-ACT =: Pooled Resource Open-Access Clinical Trial database
SD =: standard deviation
