# Supplementary material for: Multicentre appraisal of amyotrophic lateral sclerosis biofluid biomarkers shows primacy of blood neurofilament light chain
Source: Brain Commun. 2022 Feb 9;4(1):fcac029. doi: 10.1093/braincomms/fcac029 (PMC8870425; doi:10.1093/braincomms/fcac029)
Supplement: fcac029_Supplementary_Data [file fcac029_supplementary_data.docx]

**Multicentre appraisal of amyotrophic lateral sclerosis biofluid biomarkers shows primacy of blood neurofilament light chain**

**Supplementary methods**

**Clinical Laboratory Assays**

In Oxford, serum ferritin was analysed using a two-step chemiluminescent microparticle immunoassay (CMIA) using an Abbott Architext i2000 (Abbott Diagnostics, Maidenhead, UK). Plasma CK and CRP were both measured using the Abbott Architext c16000 general chemistry analyser using standard methods: CK using an enzymatic N-acetyl-L-cysteine (NAC) method and CRP a homogenous particle enhanced turbidimetric immunoassay (PETIA). CRP reproducibility was 3.4% at 3.7 mg/L and 1.9% at 29.9mg/L; CK was 1.7% at 81 U/L and 1.4% at 405 U/L. For ferritin reproducibility was 4.2% at 29.5 microg/L, 3.0% at 177.7 microg/L, 3.9% at 387.1 microg/L. In London, ferritin was measured using a Beckman Coulter Ferritin assay on the DXI platform. CK was measured using the Beckman Coulter CK-NAC assay on the AU5800 analyser. CRP was measured using the Beckman Coulter CRP Latex assay on the AU5800. CRP reproducibility was 1.9% at 6.6 mg/L and 3.3% at 64.8mg/L; CK was 1.5% at 117 U/L and 2.5% at 452 U/L. For ferritin reproducibility was 4.1% at 37.2 ng/mL, 4.3% at 118.9 ng/mL, 6.3% at 311.8 ng/mL.

Complement C3 and C4 were measured by Clinical Immunology laboratories at Oxford University Hospitals NHS Foundation Trust, Sheffield Teaching Hospitals NHS Foundation Trust and Barts Health NHS Trust, London. In Oxford, C3 and C4 were measured by turbidimetry on an Abbott Architect C4000 analyser. Interassay precision for C3 was 1.6% at 72.3, 1.2% at 227.1 and for C4 was 1.6% at 18.1 and 1.1% at 54.4.

All methods were monitored regularly using internal quality control procedures and assessed through participation in external quality assurance schemes. Samples were diluted where necessary to achieve a concentration within the linear range of standard curve measurements.

**Clinical trial modelling**

Clinical trial simulations were performed based on longitudinal linear mixed-effects models, based on the relationship between baseline PR, latency from symptom onset to enrolment and longitudinal ALSFRS-R decline and plasma NFL, with treatment effect varied by manipulating PR and latency from symptom onset to enrolment. Since recent ALS therapy trials have restricted enrolment to patients within 24 months of symptom onset, the base models upon which simulations were performed were constructed using the first 8 months of data for participants enrolled within 24 months of symptom onset (n=63 participants with longitudinal data).

ALSFRS-R scores were transformed using a Box-Cox transformation of [48-ALSFRS-R], *λ* = -0.31, yielding an approximately normal distribution centered around zero. For the simulations, PR was calculated as
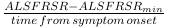


Longitudinal linear mixed-effects models were constructed using the nlme package in R. Natural log-transformed baseline PR and latency were modelled as a bivariate normal distribution, mean and standard deviation of log(baseline PR) and log(baseline latency).

Longitudinal natural log-transformed plasma NFL was modelled with fixed effects for duration from symptom onset, log(baseline PR), and time, with intercept and slope per participant random effects. For participant *j* at time *t*:


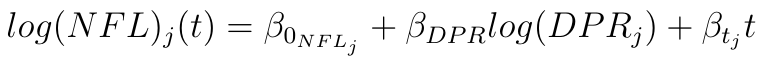

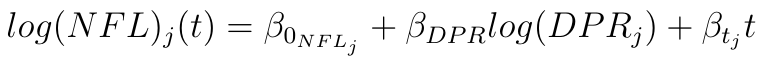

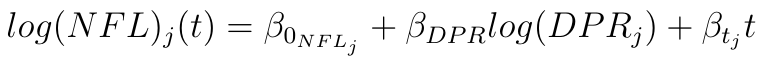


Where $\beta_{0}$ is a person’s baseline plasma NFL, $\beta_{PR}$ is the degree to which NFL reflects the person’s initial PR, and $\beta_{t}$ is the rate at which plasma NFL increases.

Longitudinal transformed ALSFRS-R was modelled with fixed effects for interactions between time and baseline PR, and per-participant intercept and time random effects, for participant *j* at time *t*:


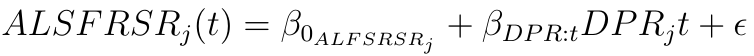

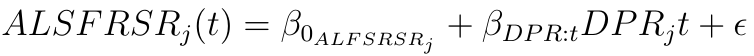

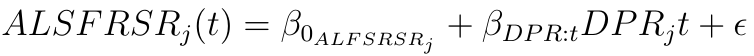


Where the first term reflects an individual’s baseline ALSFRS-R, and $\beta_{PR:t}$ is the degree to which estimated disease progress ($PR\times t$) translates into degree of disability.

Simulations of log(baseline PR) and log(baseline latency) were performed by random draws from a bivariate normal distribution, mean and standard deviation corresponding to the base models. ALSFRS-R and NFL were simulated using these baseline variables and the above longitudinal models.

Treatment effects were modelled as a change in the PR. Data from in HIV dementia suggest that plasma NFL levels fall exponentially towards normal levels over approximately 100 days from initiation of antiretroviral therapy, closely following the decline in HIV viral load. Drug effects in the treated group were therefore modelled as a proportional reduction *R* in the PR using an exponential decay:


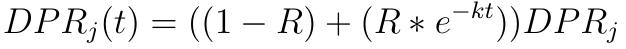

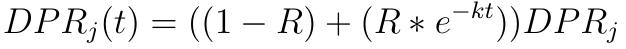


Where 1/*k* = 1 month indicates the time constant over which PR reaches its new, lower level after starting the drug. Per-participant random treatment effect was specified by random draw from a normal distribution with mean *R* and standard deviation *R*/2 and a ceiling treatment effect of 1.

The effective PR (ePR), i.e. the slope of the ALSFRS-R at time *t*:


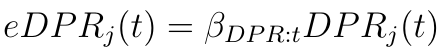

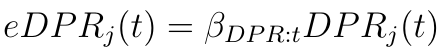

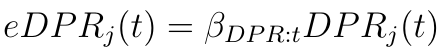

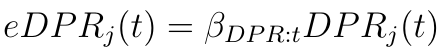


This then allows us to simulate longitudinal ALSFRS-R for participant *j* at time *t*, using this instantaneous progression rate:


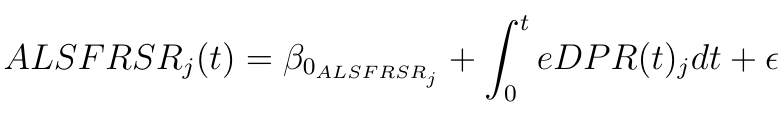

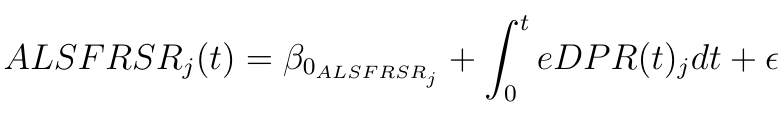


And we assume this rate is coupled NFL using the previously established relationship, giving log (plasma NFL) for participant *j* at time *t*:


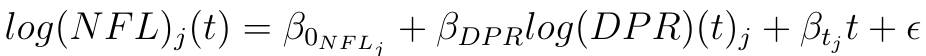

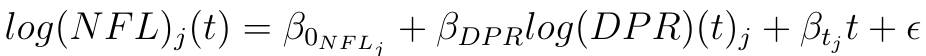

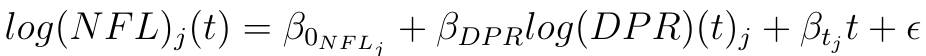


Integrals were calculated using Euler’s method. Clinical trials were modelled for different numbers of participants per group n from 6 to 120 in steps of 2 participants, using different treatment effects with follow up visits at 2 monthly intervals. Treatment effect was then measured using mixed models for repeated measures of longitudinal of untransformed ALSFRS-R data or log(plasma NFL) data, as well as ANOVA of ALSFRS-R or log(plasma NFL) difference at study end based on an intention-to treat analysis. Participant dropout was simulated using uniform attrition of 5% of participants from any visit after the first, with missing data imputation by last observation carried forward.

The proportion of iterations with p<0.05 was used to estimate the power for a given treatment effect and number of participants from 1000 iterations. These simulations estimated an untreated median ALSFRS-R decline over 6 months of 3.70 (1.14-7.13) points (IQR 1.14-7.13) compared with a median reduction of 4.94 (IQR 2.12-8.71) points in participants in the large PRO-ACT database, for ALSFRS-R scores reported at 5-7 months from enrolment, suggesting that this measure is slightly lower than previous clinical trials.^33^

|  |  |  |  |  |  |  |  |  |  |  |  |  |  |  |
| --- | --- | --- | --- | --- | --- | --- | --- | --- | --- | --- | --- | --- | --- | --- |
|  | PR | |  | Delta FRS | |  | ALSFRS-R | |  | FVC | |  | ALS-ECAS | |
|  | *r* | *p* |  | *r* | *p* |  | *r* | *p* |  | *r* | *p* |  | *r* | *p* |
| CSF NFL | **0.455** | **<0.001** |  | **0.602** | **<0.001** |  | **-0.259** | **0.032** |  | -0.241 | 0.116 |  | -0.064 | 0.894 |
| CSF CHIT1 | **0.419** | **0.005** |  | **0.479** | **0.012** |  | -0.254 | 0.063 |  | -0.287 | 0.116 |  | -0.160 | 0.894 |
| Plasma NFL | **0.476** | **<0.001** |  | **0.589** | **<0.001** |  | **-0.160** | **0.032** |  | -0.141 | 0.149 |  | -0.057 | 0.894 |
| CK | -0.094 | 0.310 |  | -0.137 | 0.258 |  | **0.182** | **0.032** |  | -0.02 | 0.805 |  | -0.034 | 0.894 |
| Ferritin | 0.081 | 0.346 |  | 0.171 | 0.17 |  | -0.054 | 0.482 |  | -0.084 | 0.367 |  | 0.114 | 0.894 |
| CRP | 0.039 | 0.647 |  | 0.042 | 0.763 |  | **-0.170** | **0.032** |  | **-0.208** | **0.047** |  | 0.006 | 0.948 |
| C3 | **0.162** | **0.045** |  | 0.185 | 0.162 |  | **-0.162** | **0.032** |  | **-0.233** | **0.034** |  | 0.114 | 0.894 |
| C4 | **0.209** | **0.008** |  | -0.100 | 0.403 |  | **-0.213** | **0.021** |  | -0.133 | 0.163 |  | 0.014 | 0.948 |
|  |  |  |  |  |  |  |  |  |  |  |  |  |  |  |
|  |  |  |  |  |  |  |  |  |  |  |  |  |  |  |

**Supplementary Table 1** Univariate clinical correlations between log-transformed analyte levels and clinical parameters. Data are Pearson *r* of log_10_ transformed analyte levels and clinical variable and FDR-adjusted p-value.

PR – baseline disease progression rate; Delta FRS – ALSFRS-R rate of decline over longitudinal visits; ALSFRS-R – revised ALS functional rating scale; FVC – forced vital capacity; ALS-ECAS – ALS-specific subscore of the Edinburgh cognitive and behavioural ALS screen; CSF – cerebrospinal fluid; NFL – neurofilament light chain; CHIT1 – chitotrisidase 1; CK – creatine kinase; CRP – C-reactive protein.

**
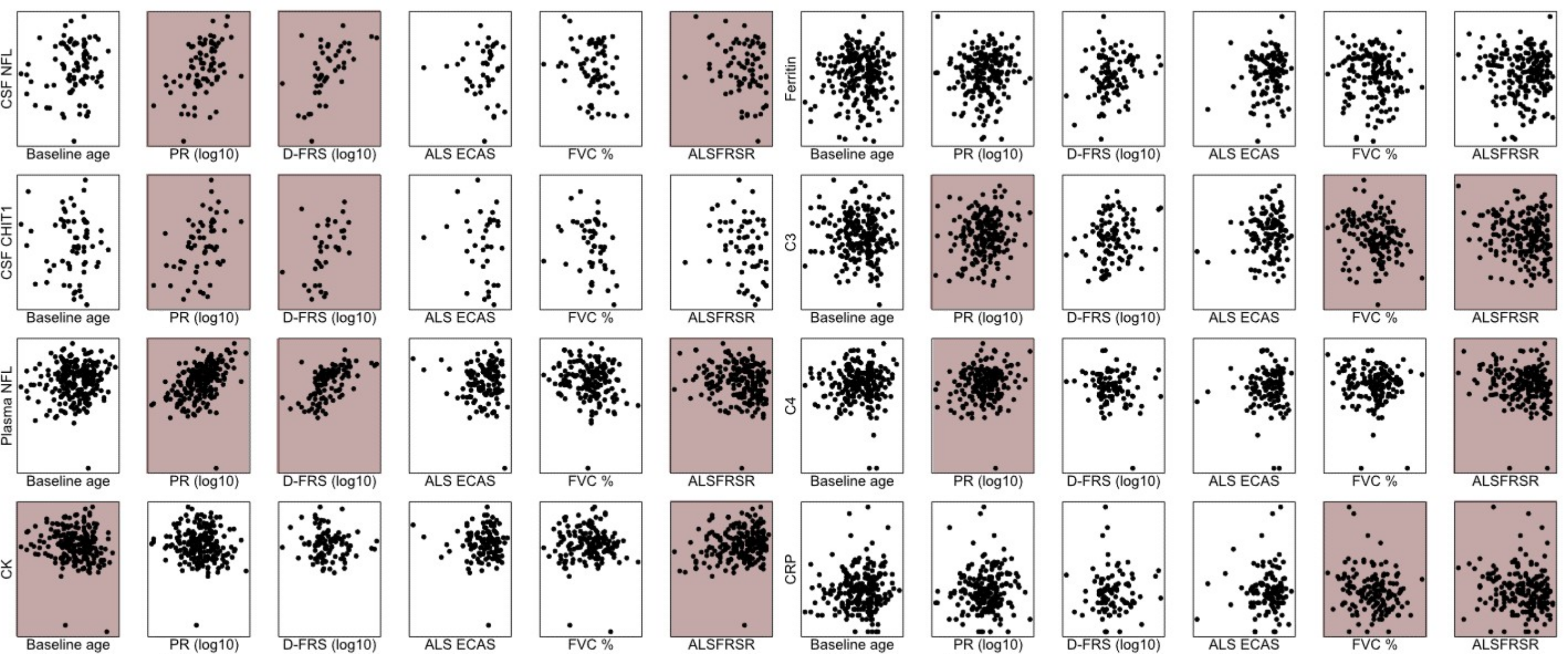
Supplementary Figure 1** Relationship between clinical variables and analyte levels. Correlation coefficients and adjusted p-values are given in Supplementary table 1. Shaded plots indicate correlations with p<0.05 after FDR correction. Analyte angles are log-transformed.

PR – baseline disease progression rate; D-FRS – ALSFRS-R rate of decline over longitudinal visits; ALSFRS-R – revised ALS functional rating scale; FVC – forced vital capacity; ALS-ECAS – ALS-specific subscore of the Edinburgh cognitive and behavioural ALS screen; CSF – cerebrospinal fluid; NFL – neurofilament light chain; CHIT1 – chitotriosidase 1; CK – creatine kinase; CRP – C-reactive protein.

|  |  |  |  |  |  |  |  |  |  |  |  |  |  |  |  |  |  |  |  |  |  |  |  |
| --- | --- | --- | --- | --- | --- | --- | --- | --- | --- | --- | --- | --- | --- | --- | --- | --- | --- | --- | --- | --- | --- | --- | --- |
|  | CSF NFL | |  | CSF CHIT1 | |  | Plasma NFL | |  | CK | |  | Ferritin | |  | CRP | |  | C3 | |  | C4 | |
|  | Slope | p |  | Slope | p |  | Slope | p |  | Slope | p |  | Slope | p |  | Slope | p |  | Slope | p |  | Slope | p |
| Log_10_[analyte] | **0.449** | **<0.001** |  | **0.371** | **0.023** |  | **0.579** | **<0.001** |  | -0.063 | 0.566 |  | 0.140 | 0.177 |  | 0.033 | 0.652 |  | 0.947 | 0.056 |  | **0.549** | **0.048** |
| Sex | -0.031 | 0.736 |  | -0.002 | 0.989 |  | -0.035 | 0.546 |  | -0.079 | 0.247 |  | -0.114 | 0.104 |  | -0.087 | 0.197 |  | -0.108 | 0.099 |  | -0.084 | 0.205 |
| Cognitive onset | -0.353 | 0.363 |  | -0.358 | 0.409 |  | -0.206 | 0.365 |  | -0.292 | 0.198 |  | -0.258 | 0.254 |  | -0.278 | 0.225 |  | -0.355 | 0.112 |  | -0.378 | 0.092 |
| Spinal onset | -0.209 | 0.071 |  | -0.203 | 0.145 |  | 0.04 | 0.521 |  | -0.097 | 0.187 |  | -0.05 | 0.483 |  | -0.074 | 0.315 |  | -0.124 | 0.079 |  | -0.109 | 0.118 |
| Age at sampling | 0.002 | 0.534 |  | 0.001 | 0.780 |  | -0.002 | 0.483 |  | -0.001 | 0.722 |  | 0.000 | 0.914 |  | 0.000 | 0.943 |  | 0.000 | 0.922 |  | -0.001 | 0.841 |
| Adjusted R^2^ | 0.235 | |  | 0.135 | |  | 0.219 | |  | 0.005 | |  | 0.004 | |  | -0.005 | |  | 0.031 | |  | 0.034 | |
| n | 74 | |  | 56 | |  | 206 | |  | 199 | |  | 203 | |  | 200 | |  | 198 | |  | 198 | |
|  |  |  |  |  |  |  |  |  |  |  |  |  |  |  |  |  |  |  |  |  |  |  |  |

**Supplementary Table 2** Multiple linear regression, modelling PR as the dependent variable, log-transformed analyte level, sex, onset site and age at sampling as independent variables. Analyte p-values have been FDR adjusted.

PR – baseline disease progression rate; CSF – cerebrospinal fluid; NFL – neurofilament light chain; CHIT1 – chitotrisidase 1; CK – creatine kinase; CRP – C-reactive protein

|  |  |  |  |  |  |  |  |  |  |  |  |  |  |  |  |  |  |  |  |  |  |  |  |
| --- | --- | --- | --- | --- | --- | --- | --- | --- | --- | --- | --- | --- | --- | --- | --- | --- | --- | --- | --- | --- | --- | --- | --- |
|  | CSF NFL | |  | CSF CHIT1 | |  | Plasma NFL | |  | CK | |  | Ferritin | |  | CRP | |  | C3 | |  | C4 | |
|  | Slope | p |  | Slope | p |  | Slope | p |  | Slope | p |  | Slope | p |  | Slope | p |  | Slope | p |  | Slope | p |
| Log_10_[analyte] | **0.227** | **<0.001** |  | **0.140** | **0.027** |  | **0.256** | **<0.001** |  | -0.033 | 0.703 |  | 0.089 | 0.064 |  | 0.015 | 0.733 |  | 0.446 | 0.062 |  | -0.147 | 0.325 |
| Sex | -0.025 | 0.581 |  | -0.010 | 0.830 |  | -0.007 | 0.782 |  | -0.015 | 0.660 |  | -0.030 | 0.381 |  | -0.017 | 0.615 |  | -0.022 | 0.509 |  | -0.020 | 0.553 |
| Cognitive onset | - | - |  | - | - |  | - | - |  | -0.013 | 0.929 |  | -0.005 | 0.973 |  | -0.038 | 0.802 |  | -0.051 | 0.726 |  | -0.008 | 0.956 |
| Spinal onset | 0.032 | 0.565 |  | -0.025 | 0.645 |  | 0.003 | 0.899 |  | -0.040 | 0.257 |  | -0.041 | 0.210 |  | -0.053 | 0.130 |  | -0.060 | 0.071 |  | -0.049 | 0.140 |
| Age at sampling | -0.002 | 0.428 |  | 0.002 | 0.436 |  | -0.001 | 0.427 |  | -0.001 | 0.544 |  | -0.001 | 0.457 |  | -0.001 | 0.673 |  | -0.001 | 0.621 |  | -0.001 | 0.583 |
| Adjusted R^2^ | 0.29 | |  | 0.146 | |  | 0.321 | |  | -0.017 | |  | 0.02 | |  | -0.02 | |  | 0.033 | |  | -0.001 | |
| n | 74 | |  | 56 | |  | 206 | |  | 199 | |  | 203 | |  | 200 | |  | 198 | |  | 198 | |
|  |  |  |  |  |  |  |  |  |  |  |  |  |  |  |  |  |  |  |  |  |  |  |  |

**Supplementary Table 3** Multiple linear regression, modelling delta-ALSFRSR as the dependent variable, log-transformed analyte level, sex, onset site and age at sampling as independent variables. Analyte p-values have been FDR adjusted.

PR – baseline disease progression rate; CSF – cerebrospinal fluid; NFL – Neurofilament light chain; CHIT1 – chitotrisidase 1; CK – creatine kinase; CRP – C-reactive protein

|  |  |  |  |  |  |  |  |  |  |  |  |  |  |  |  |  |  |  |  |  |  |  |  |
| --- | --- | --- | --- | --- | --- | --- | --- | --- | --- | --- | --- | --- | --- | --- | --- | --- | --- | --- | --- | --- | --- | --- | --- |
|  | CSF NFL | |  | CSF CHIT1 | |  | Plasma NFL | |  | CK | |  | Ferritin | |  | CRP | |  | C3 | |  | C4 | |
|  | Slope | p |  | Slope | p |  | Slope | p |  | Slope | p |  | Slope | p |  | Slope | p |  | Slope | p |  | Slope | p |
| Log_10_[analyte] | -3.164 | 0.119 |  | -2.516 | 0.233 |  | **-3.873** | **0.050** |  | 3.194 | 0.063 |  | -1.664 | 0.233 |  | -1.947 | 0.115 |  | -14.64 | 0.098 |  | **-9.228** | **0.050** |
| Sex | 2.128 | 0.172 |  | 1.16 | 0.533 |  | 0.992 | 0.353 |  | 1.101 | 0.309 |  | 2.092 | 0.066 |  | 1.438 | 0.183 |  | 1.754 | 0.101 |  | 1.449 | 0.175 |
| Cognitive onset | -6.315 | 0.336 |  | -6.771 | 0.309 |  | -0.223 | 0.957 |  | -0.398 | 0.913 |  | -0.381 | 0.918 |  | -0.274 | 0.941 |  | 0.727 | 0.843 |  | 1.228 | 0.737 |
| Spinal Onset | 0.942 | 0.626 |  | 1.045 | 0.622 |  | -2.669 | 0.021 |  | -1.918 | 0.104 |  | -1.498 | 0.201 |  | -1.163 | 0.322 |  | -1.034 | 0.369 |  | -1.304 | 0.25 |
| Age at sampling (years) | -0.066 | 0.305 |  | -0.061 | 0.408 |  | -0.073 | 0.097 |  | -0.047 | 0.29 |  | -0.067 | 0.134 |  | -0.067 | 0.127 |  | -0.062 | 0.158 |  | -0.052 | 0.236 |
| Adjusted R^2^ | 0.069 | |  | 0.017 | |  | 0.049 | |  | 0.029 | |  | 0.017 | |  | 0.022 | |  | 0.025 | |  | 0.038 | |
| n | 76 | |  | 58 | |  | 211 | |  | 203 | |  | 207 | |  | 205 | |  | 201 | |  | 201 | |
|  |  |  |  |  |  |  |  |  |  |  |  |  |  |  |  |  |  |  |  |  |  |  |  |

**Supplementary Table 4** Multiple linear regression, modelling ALSFRS-R as the dependent variable, log-transformed analyte level, sex, onset site and age at sampling as independent variables. Analyte p-values have been FDR adjusted.

ALSFRS-R – revised ALS functional rating scale; CSF – cerebrospinal fluid; NFL – neurofilament light chain; CHIT1 – chitotriosidase 1; CK – creatine kinase; CRP – C-reactive protein

|  |  |  |  |  |  |  |  |  |  |  |  |  |  |  |  |  |  |  |  |  |
| --- | --- | --- | --- | --- | --- | --- | --- | --- | --- | --- | --- | --- | --- | --- | --- | --- | --- | --- | --- | --- |
|  | CSF NFL | |  | Plasma NFL | |  | CK | |  | Ferritin | |  | CRP | |  | C3 | |  | C4 | |
|  | Slope | p |  | Slope | p |  | Slope | p |  | Slope | p |  | Slope | p |  | Slope | p |  | Slope | p |
| Log_10_[analyte] | -3.806 | 0.816 |  | -1.316 | 0.879 |  | -2.640 | 0.816 |  | 5.192 | 0.816 |  | -1.893 | 0.816 |  | -12.33 | 0.816 |  | 13.55 | 0.816 |
| Sex | -5.960 | 0.267 |  | -12.77 | 0.001 |  | -11.76 | 0.002 |  | -14.34 | <0.001 |  | -12.35 | 0.001 |  | -13.39 | <0.001 |  | -12.73 | 0.001 |
| Cognitive onset | - | - |  | 0.062 | 0.998 |  | -12.68 | 0.4 |  | -12.95 | 0.368 |  | -13.26 | 0.378 |  | -11.71 | 0.428 |  | -14.12 | 0.337 |
| Spinal Onset | -2.497 | 0.722 |  | 6.459 | 0.127 |  | 9.313 | 0.041 |  | 9.619 | 0.022 |  | 9.447 | 0.033 |  | 9.98 | 0.020 |  | 9.568 | 0.023 |
| Age at sampling (years) | 0.471 | 0.035 |  | 0.149 | 0.329 |  | 0.192 | 0.206 |  | 0.23 | 0.116 |  | 0.198 | 0.196 |  | 0.196 | 0.200 |  | 0.224 | 0.129 |
| Disease duration at sampling (months) | 0.167 | 0.365 |  | -0.038 | 0.639 |  | -0.064 | 0.394 |  | -0.037 | 0.609 |  | -0.063 | 0.398 |  | -0.058 | 0.436 |  | -0.045 | 0.538 |
| ALSFRS-R score | 2.522 | <0.001 |  | 2.477 | <0.001 |  | 2.6 | <0.001 |  | 2.629 | <0.001 |  | 2.564 | <0.001 |  | 2.522 | <0.001 |  | 2.626 | <0.001 |
| Adjusted R^2^ | 0.364 | |  | 0.429 | |  | 0.444 | |  | 0.464 | |  | 0.447 | |  | 0.447 | |  | 0.451 | |
| n | 59 | |  | 134 | |  | 130 | |  | 133 | |  | 129 | |  | 130 | |  | 130 | |
|  |  |  |  |  |  |  |  |  |  |  |  |  |  |  |  |  |  |  |  |  |

**Supplementary Table 5** Multiple linear regression, modelling FVC as the dependent variable, log-transformed analyte level, sex, onset site, age at sampling, disease duration and ALSFRS-R as independent variables. Analyte p-values have been FDR adjusted.

FVC – forced vital capacity; ALSFRS-R – revised ALS functional rating scale; FVC – forced vital capacity; CSF – cerebrospinal fluid; NFL – neurofilament light chain; CHIT1 – chitotriosidase 1; CK – creatine kinase; CRP – C-reactive protein

|  |  |  |  |  |  |  |  | |  | |  |  | |  | |  |  | |  | |  |  | |  | |  |  | |  | |  |  | |  | |  |  |  |  |  |
| --- | --- | --- | --- | --- | --- | --- | --- | --- | --- | --- | --- | --- | --- | --- | --- | --- | --- | --- | --- | --- | --- | --- | --- | --- | --- | --- | --- | --- | --- | --- | --- | --- | --- | --- | --- | --- | --- | --- | --- | --- |
|  | CSF NFL | |  | CSF CHIT1 | |  | Plasma NFL | | | |  | CK | | | |  | Ferritin | | | |  | CRP | | | |  | C3 | | | |  | C4 | | | |  |  |  |  |  |
|  | Slope | p |  | Slope | p |  | Slope | | p | |  | Slope | | p | |  | Slope | | p | |  | Slope | | p | |  | Slope | | p | |  | Slope | | p | |  |  |  |  |  |
| Log_10_[analyte] | -3.402 | 0.769 |  | -5.042 | 0.769 |  | -1.301 | | 0.769 | |  | -4.493 | | 0.769 | |  | 1.739 | | 0.769 | |  | -1.215 | | 0.769 | |  | 13.08 | | 0.769 | |  | -3.895 | | 0.769 | |  |  |  |  |  |
| Sex | 7.344 | 0.232 |  | 6.607 | 0.391 |  | 2.868 | | 0.357 | |  | 3.268 | | 0.316 | |  | 3.613 | | 0.252 | |  | 2.677 | | 0.416 | |  | 3.765 | | 0.229 | |  | 3.462 | | 0.276 | |  |  |  |  |  |
| Cognitive onset | - | - |  | - | - |  | 7.783 | | 0.487 | |  | 9.593 | | 0.403 | |  | 3.615 | | 0.737 | |  | 6.083 | | 0.606 | |  | 4.175 | | 0.702 | |  | 5.311 | | 0.626 | |  |  |  |  |  |
| Spinal onset | -4.324 | 0.641 |  | -5.352 | 0.596 |  | 1.619 | | 0.613 | |  | 5.365 | | 0.135 | |  | -0.743 | | 0.814 | |  | 3.327 | | 0.326 | |  | -0.441 | | 0.894 | |  | 0.096 | | 0.977 | |  |  |  |  |  |
| Age at sampling (years) | 0.139 | 0.511 |  | 0.084 | 0.76 |  | 0.155 | | 0.141 | |  | 0.102 | | 0.334 | |  | 0.112 | | 0.265 | |  | 0.1 | | 0.355 | |  | 0.123 | | 0.244 | |  | 0.099 | | 0.327 | |  |  |  |  |  |
| Disease duration at sampling (months) | -0.626 | 0.075 |  | -0.622 | 0.132 |  | -0.091 | | 0.109 | |  | -0.033 | | 0.531 | |  | -0.052 | | 0.284 | |  | -0.041 | | 0.427 | |  | -0.046 | | 0.354 | |  | -0.054 | | 0.276 | |  |  |  |  |  |
| ALSFRS-R score | -0.966 | 0.196 |  | -1.149 | 0.214 |  | -0.433 | | 0.106 | |  | -0.129 | | 0.65 | |  | -0.396 | | 0.143 | |  | -0.205 | | 0.49 | |  | -0.395 | | 0.168 | |  | -0.478 | | 0.088 | |  |  |  |  |  |
| FVC | 0.13 | 0.41 |  | 0.129 | 0.502 |  | 0.038 | | 0.551 | |  | 0.008 | | 0.903 | |  | 0.077 | | 0.231 | |  | 0.006 | | 0.932 | |  | 0.087 | | 0.178 | |  | 0.09 | | 0.167 | |  |  |  |  |  |
| Adjusted R^2^ | -0.013 | |  | -0.11 | |  | 0.005 | | | |  | -0.02 | | | |  | -0.023 | | | |  | -0.053 | | | |  | -0.017 | | | |  | -0.021 | | | |  |  |  |  |  |
| n | 30 | |  | 20 | |  | 57 | | | |  | 58 | | | |  | 58 | | | |  | 57 | | | |  | 58 | | | |  | 58 | | | |  |  |  |  |  |
|  |  |  |  |  |  |  | |  | |  |  | |  | |  |  | |  | |  |  | |  | |  |  | |  | |  |  | |  | |  |  |  |  |  |  |

**Supplementary Table 6** Multiple linear regression, modelling ALS-specific ECAS score as the dependent variable, log-transformed analyte level, sex, onset site, age at sampling, disease duration, ALSFRS-R and FVC as independent variables. Analyte p-values have been FDR adjusted.

ECAS – Edinburgh cognitive and behavioural ALS screen; ALSFRS-R – revised ALS functional rating scale; FVC – forced vital capacity; CSF – cerebrospinal fluid; NFL – neurofilament light chain; CHIT1 – chitotriosidase 1; CK – creatine kinase; CRP – C-reactive protein


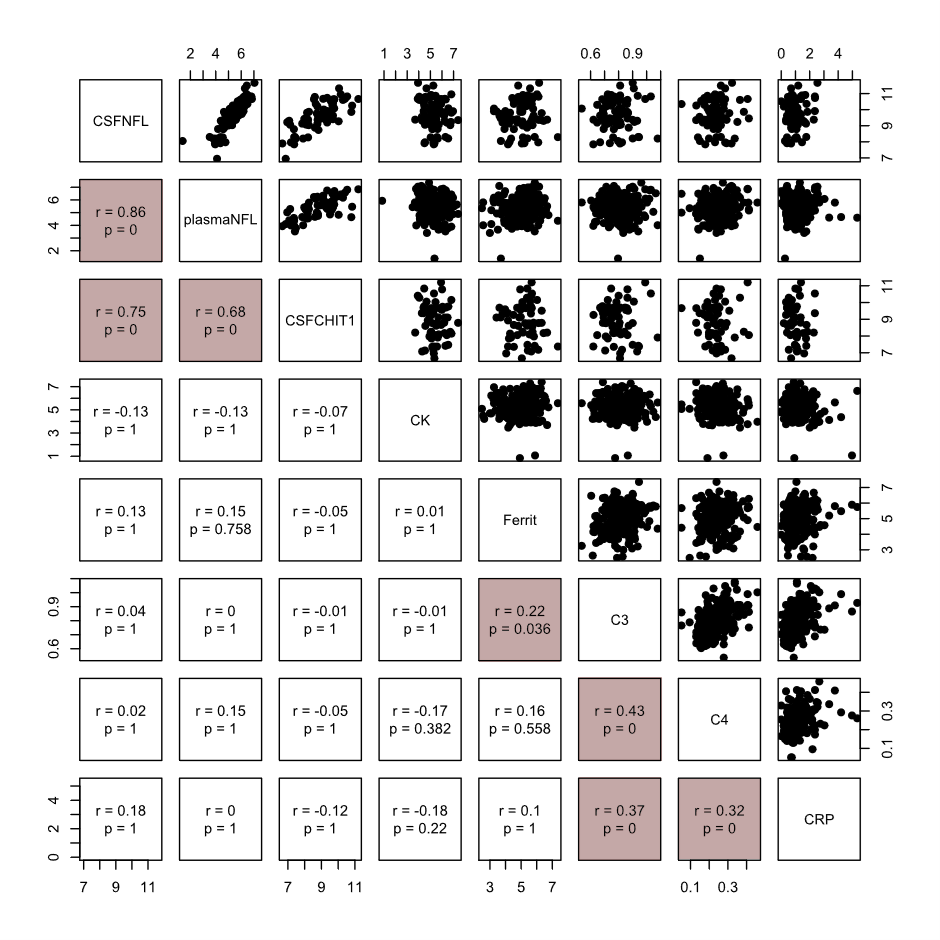


**Supplementary Figure 2** Correlations between log-transformed analyte levels. Data are Pearson r and FDR-adjusted p. Shaded cells highlight significant correlations.

CSF – cerebrospinal fluid; NFL – neurofilament light chain; CHIT1 – chitotriosidase 1; CK – creatine kinase; CRP – C-reactive protein; Ferrit – Ferritin

**
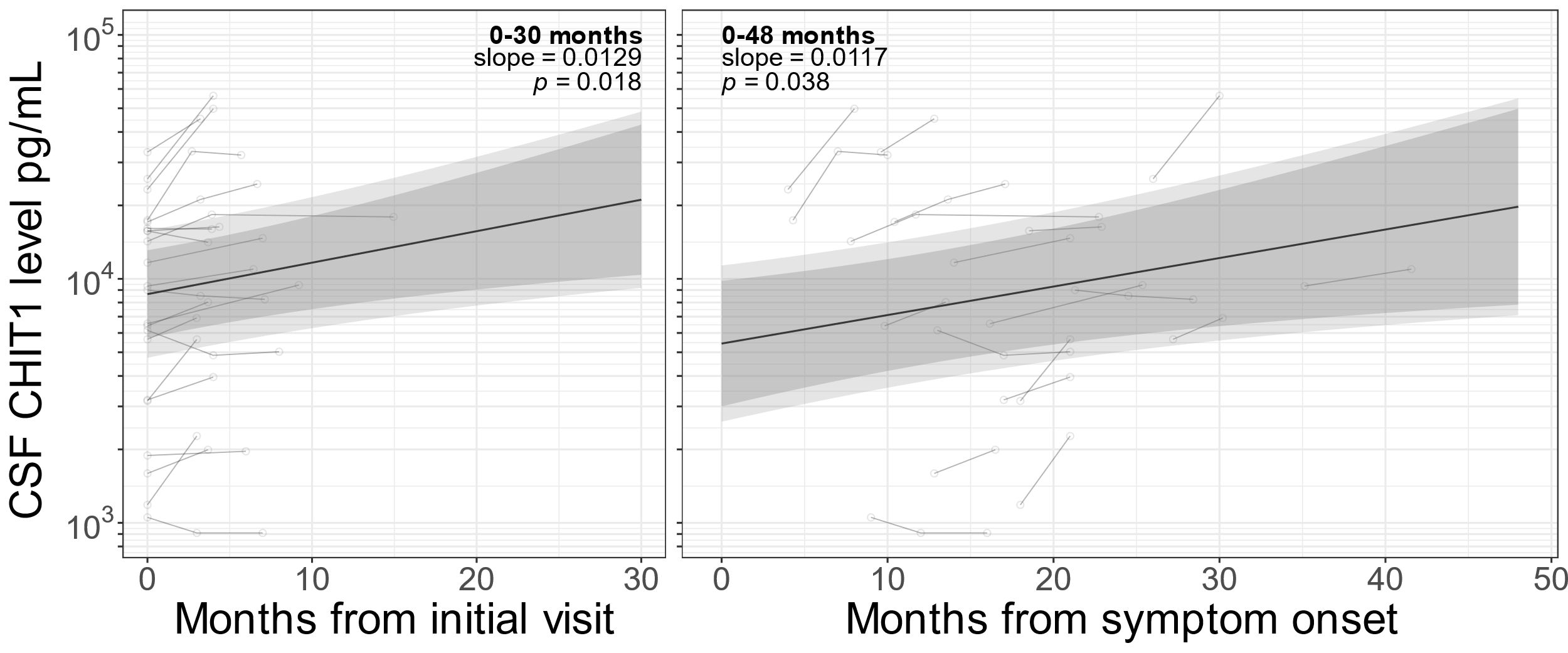
**

**B**

**A**

**Supplementary Figure 3** Longitudinal analysis of CSF CHIT1 of patients with ALS. Linear mixed effects models were fitted to data from **A** baseline visit and **B** symptom onset.

CSF – cerebrospinal fluid; ALS – amyotrophic lateral sclerosis; CHIT1 – chitotriosidase 1.


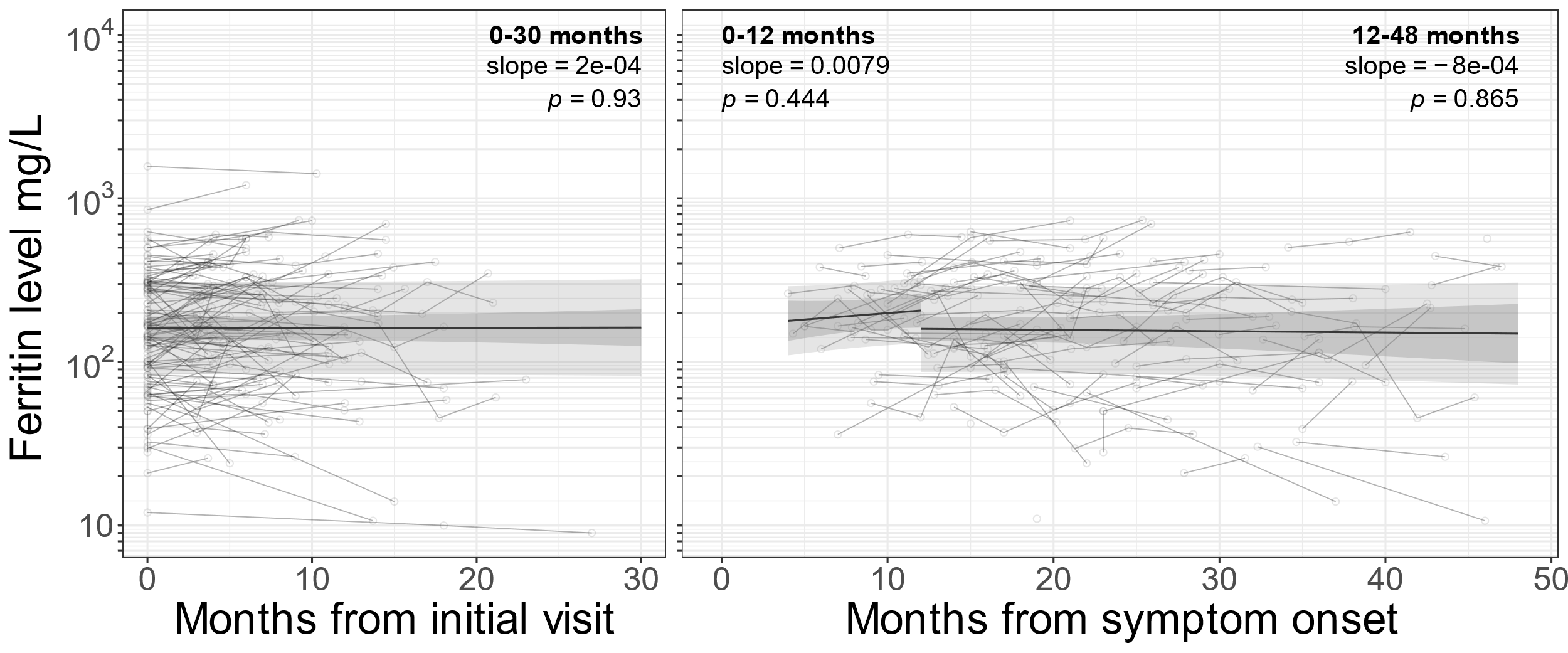

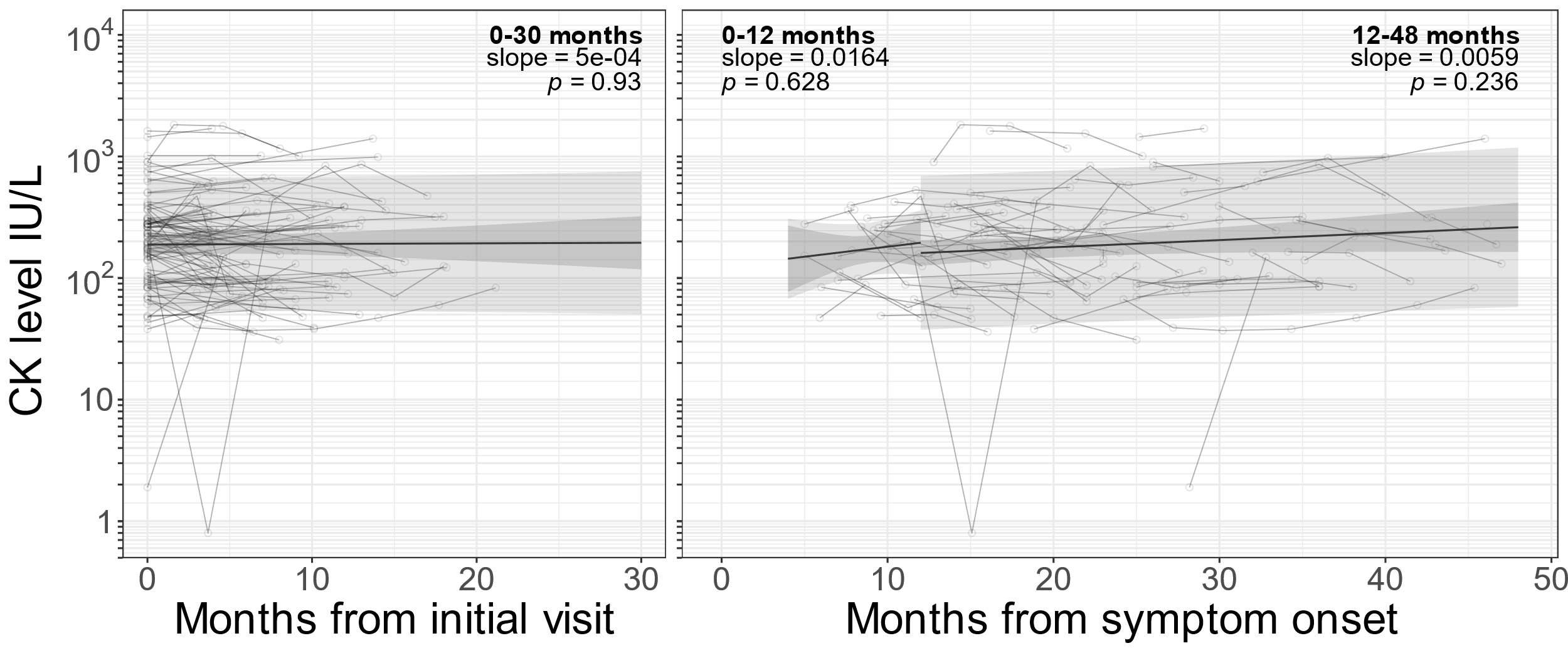

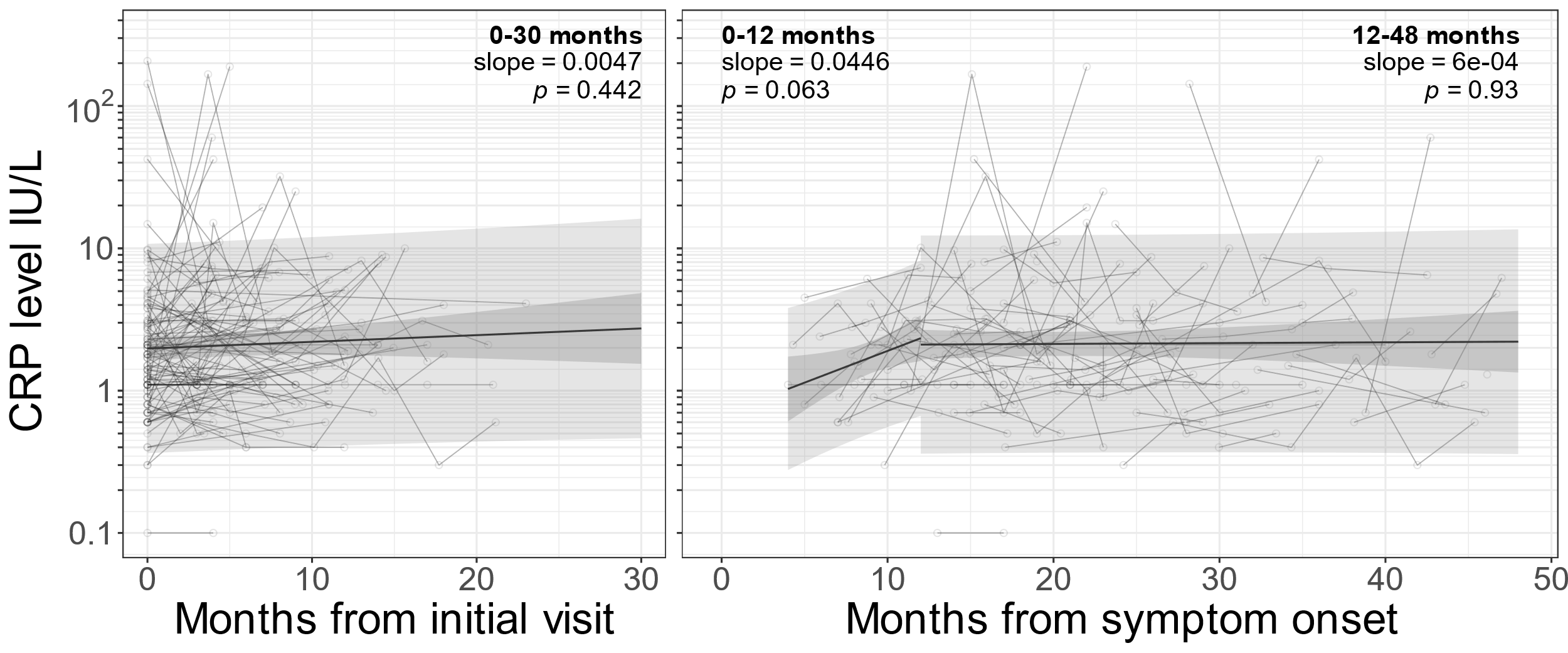

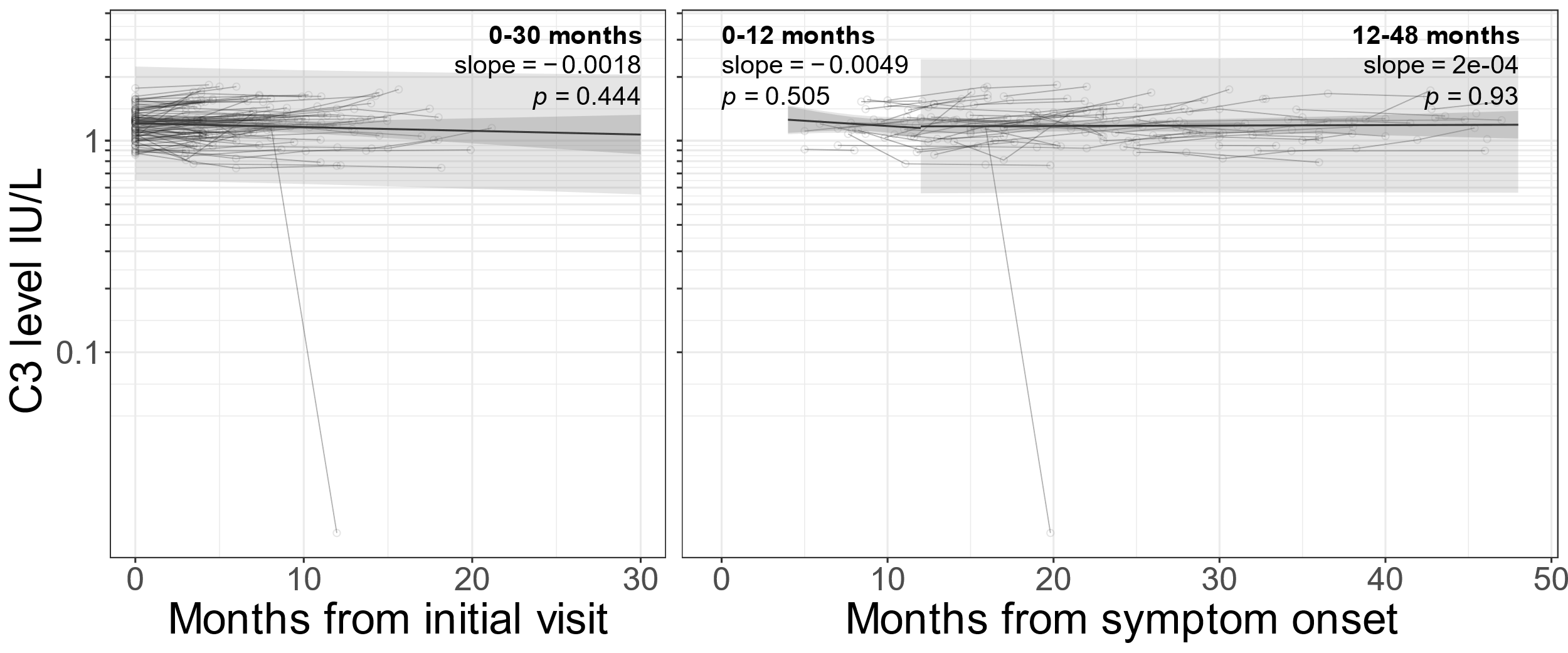
**
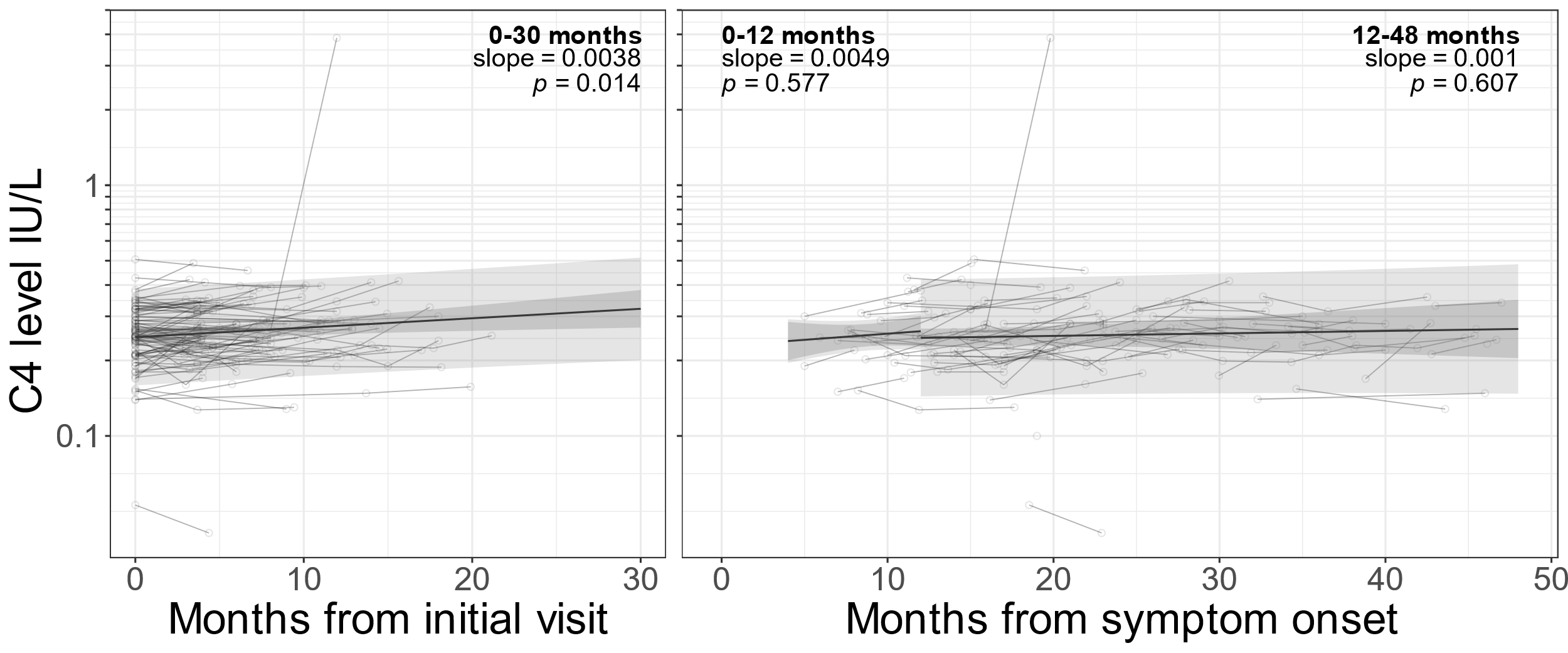
**

**H**

**G**

**I**

**E**

**F**

**J**

**D**

**C**

**B**

**A**

**Supplementary Figure 4** Longitudinal analysis of blood analytes ferritin (**A**-**B**), CK (**C**-**D**), CRP (**E**-**F**), complement C3 (**G**-**H**), complement C4 (**I**-**J**) of patients with ALS. Linear mixed effects models were fitted to data from baseline visit (**A**, **C**, **E**, **G**, **I**) and from symptom onset (**B**, **D**, **F**, **H**, **J**). Separate models of plasma neurofilament from symptom onset were constructed for samples taken within 12 months of symptom onset and those 12-48 months from symptom onset.

ALS – amyotrophic lateral sclerosis; CK – creatine kinase; CRP – C-reactive protein.

| Diagnosis | n |
| --- | --- |
| Nonprogressive monomelic amyotrophy | 10 |
| Spastic paraparesis | 6 |
| Multiple sclerosis / clinically isolated syndrome | 6 |
| Conduction block neuropathy | 4 |
| Peripheral neuropathy | 4 |
| Spondylosis | 2 |
| Lumbosacral radiculopathy | 2 |
| Kennedy's disease | 2 |
| Myasthenia gravis | 2 |
| Congenital myopathy | 2 |
| Phrenic neuropathy | 2 |
| Parkinson's disease | 2 |
| Progressive supranuclear palsy | 2 |
| Diabetic amyotrophy | 1 |
| Benign fasciculations | 1 |
| Cerebrovascular disease | 1 |
| Frontotemporal dementia | 1 |
| Central cord syndrome | 1 |
| Functional neurological disorder | 1 |
| Hereditary neuropathy with liability to pressure palsy | 1 |
| Hypoglossal neuropathy | 1 |
| Gait impairment | 1 |
| Spinocerebellar ataxia | 1 |
| Spinal muscular atrophy | 1 |
| Headache | 1 |
| Segmental myoclonus | 1 |
| Idiopathic hypersomnia | 1 |
| Narcolepsy | 1 |
| Unknown | 19 |

**Supplementary table 7** Diagnosis of disease controls seen in regional MND clinics and included in analysis.
